# Predictors for weight loss after Roux-en-Y gastric bypass: the trend and associated factors for weight loss

**DOI:** 10.1186/s12893-022-01760-3

**Published:** 2022-08-11

**Authors:** Foolad Eghbali, Mansour Bahardoust, Abdolreza Pazouki, Gelayol Barahman, Adnan Tizmaghz, Amir Hajmohammadi, Reza Karami, Fatemeh Sadat Hosseini-Baharanchi

**Affiliations:** 1 Minimally Invasive Surgery Research Center, Rasool-E Akram Hospital, Iran University of Medical Sciences, Tehran, Iran; 2 Department of Epidemiology, School of Public Health, Shahid Beheshti University of Medical Sciences, Tehran, 1449614535 Iran; 3 Medical Doctor, Islamic Azad University of Medical Sciences, Tehran, Iran; 4 Department of Biostatistics, School of Public Health, Iran University of Medical Sciences, Tehran, 1449614535 Iran

**Keywords:** Roux-en-Y gastric bypass, Weight loss, Bariatric surgery

## Abstract

**Background:**

Historically, Roux-en-Y gastric bypass (RYGB) has been considered the gold standard of bariatric surgery (BS). This procedure acts as a mixed restrictive and malabsorptive operation.

**Methods:**

This retrospective cohort study included 410 morbidly obese patients (BMI > 40 kg/m^2^ or BMI > 35 kg/m^2^ along with at least one major comorbidity) who underwent primary laparoscopic RYGB surgery from 2009 to 2015 by a single surgery team. The patients were 18 years and older with at least 12 months of follow-up. Total weight loss (%TWL) and comorbidity resolution were compared in short-term (12 months) and mid-term (12–60 months) follow-ups. The primary and secondary outcomes were evaluating the effect of Roux-en-Y on weight loss and control of comorbidities, respectively.

**Results:**

The mean ± SD age, weight, and BMI at surgery were 40.1 ± 10.58 years, 123.32 ± 19.88 kg, and 45.78 ± 5.54 kg/m^2^, respectively, and 329 (80%) were female, and 62 (15%) had T2DM. %TWL was significantly higher in T2DM patients 9 months postoperatively and after that. Patients with lower BMI (< 50 kg/m^2^) at surgery and non-diabetic patients had a significantly lower %TWL over a short- and long-term follow-up (P < 0.001).

**Conclusions:**

BS remains the most efficacious and durable weight loss treatment. However, a proportion of patients will experience insufficient weight loss following BS.

## Introduction

Roux-en-Y Gastric Bypass (RYGB) has been considered the gold standard of BS for the past two decades. It represents the second most performed procedure [1, 2]. This popularity is due to consistently satisfying and long-lasting weight loss and comorbidity resolution with acceptable complications and mortality rates [3]. Most patients expect to lose more than 70% of their excess weight in the first 12 months after the surgery. Mean %TWL is 32% at 1–2 years, then decreases slightly to 25% at 10 years and maintains this up to 20 years post-op [4]. Many clinical trials that compared different limb lengths of gastric bypass have not shown significant differences in weight loss [5]. Despite excellent weight loss, a percentage of patients still fail to lose 50% excess weight loss or reach a BMI of less than 35 kg/m^2^. The prevalence of weight loss failure following RYGB is between 5 and 40% [6, 7]. For patients with BMI > 50 kg/m^2^, acceptable weight loss may be achieved with a final BMI remaining over 35 kg/m^2^.However, there is no consensus on the definition of weight loss failure in the literature [8]. The etiology of weight regain, and weight loss failure tends to be multifactorial, including pre-operative BMI, nutritional habits, mental health and anatomical changes such as dilation of gastrojejunal anastomosis and presence of a gastro-gastric fistula [9–11]. There is a gradual tendency to regain weight over time, according to the severity of obesity. Individuals with a BMI < 50 kg/m^2^ are more likely to lose a higher percentage of their excess weight initially but tend to regain weight, similar to the patients with BMI > 50 [12]. Standardizing the report's calculation of %TWL is considered the method of choice to describe weight loss and regain after surgery [13–15]. Recent studies also suggest that the success of weight loss after BS depends on some patients’ characteristics before the surgery, including age, gender, weight, BMI, fat percentage and fat distribution. So that the younger, lower BMI, lower body fat percentage, and android fat distribution phenotype of bariatric surgery candidates probably have more successful weight loss [16–19]. This study aims to determine the associated factors related to weight loss after RYGB during 5 years follow-up.

## Methods

### Studied sample

This retrospective cohort study included 410 morbidly obese patients (BMI > 40 kg/m^2^ or BMI > 35 kg/m^2^ along with at least one obesity-related comorbidity) who underwent primary laparoscopic RYGB surgery from May 2009 to January 2015 by a single surgery team in the center of excellence. The patients were 18 years and older with at least 12 months of follow-up. The data from converted, reversed, and revised patients due to weight loss failure were included in the analysis until the failure and afterward were excluded. The cases of pregnancy after the surgery were excluded. Data were provided from the National Obesity Surgery Database, Iran. Written informed consent was obtained from all patients. The ethics committee approval code for this study is IR.IUMS.REC 1396.32051.

### Studied factors

Age, sex, preoperative BMI (categorized as 35–50, ≥ 50), and patients who reported major comorbidities at the first visit, including hypertension, type 2 diabetes mellitus (T2DM), dyslipidemia, and hypothyroidism, were included. Hypertension was defined as systolic blood pressure ≥ 140 mmHg or diastolic blood pressure ≥ 90 mmHg. T2DM was defined as fasting blood glucose ≥ 6·1 mmol/l [20]. % TWL = (pre-surgery BMI − post-surgery BMI at the time of measurement)/pre-surgery BMI × 100 at 1, 3, 6, 9, 12, 18, 24, 36, 48, and 60 months were the main outcomes of the study (in which ideal weight is defined by the weight corresponding to a BMI of 25 kg/m^2^. Short-term (12–36 months) and mid-term (36–60 months) follow-ups were the studied phases. The primary outcome was evaluating the effect of Roux-en-Y on weight loss. The secondary outcome was the effect of Roux-en-Y on the control of comorbidities. Patients whose hypothyroidism was not controlled were excluded from the study.

### Surgical technique

The patient was placed in a supine position with split legs, and the surgeon stood between the patient’s legs (French position) while inserting five trocars, and the assistant stayed on the left side. The operating table was placed in a reverse Trendelenburg position. The angle of His was initially dissected, and the left crus of the diaphragm was exposed. The anesthesiologist inserted a 36 French orogastric tube to calibrate the gastric pouch. The dissection of vascular arcades began 6 cm below the gastroesophageal junction on the lesser curvature.

Once the gastric pouch had been created using staplers, the omentum and transverse mesocolon were lifted upwards until Treitz’s ligament was identified. If the omentum were very thick, it would be divided longitudinally up to the transverse colon. The biliary limb was measured distal to the Treitz ligament. Then a side-to-side gastrojejunostomy was performed using a linear 30-mm stapler. Starting at this level, the alimentary limb was measured, and jejunojejunostomy was carried out between the dietary and biliary limbs. The remaining anastomotic defects were closed using absorbable 2-0 PDS sutures. Finally, the biliary loop and alimentary loop were separated using a linear cutter stapler. Jejunojejunal mesentery and Petersen’s space defects were closed at the end of the procedure. The mesenteric defects were closed using 2-0 non-absorbable polypropylene sutures.

### Statistical analysis

Variables are summarized using mean ± SD and frequency (%) for quantitative and qualitative variables, respectively. Patients with at least two weight measurements before the 12th month were recruited for short-term analysis. In addition, if a patient had at least two weight measures until 36 months were included in the mid-term analysis. A univariate linear mixed effect model was used to assess the effect of variables on weight loss outcomes considering random intercept and random slope [15]. The multiple linear mixed models included factors with P < 0.2 in univariate analysis. The results were reported using an estimate (95% CI: confidence interval). All the regression models are fitted to each phase of weight loss, short and mid-term. The data were analyzed using R3.5.1. P-values less than 0.05 were considered significant.

## Results

**Fig. 1:**
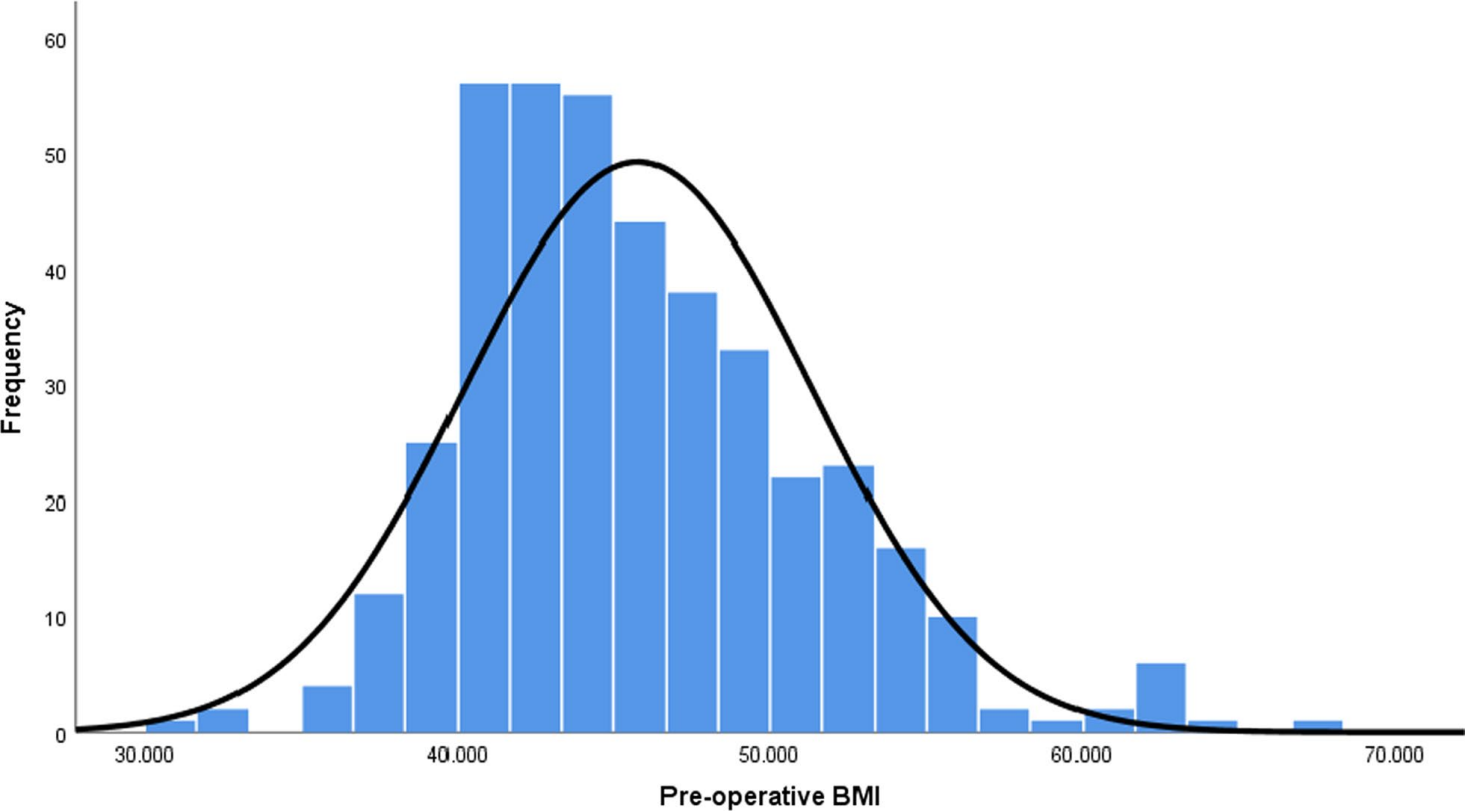
Diagram for the BMI distribution of the patients preoperatively

Four hundred and ten patients were analyzed. The mean age and weight were 40.1 ± 10.58 years, 123.32 ± 19.88 kg, respectively, and The mean initial BMI was 45.78 ± 5.54 kg/m^2^. The median (IQR) BMI was 44.28(41.03, 48.2) kg/m^2^. (Fig. 1) 329 (80%) were female, and 62 (15%) had T2DM. The median (IQR) follow-up time was 22.11 (16.8, 29.84) months. The mean number of weight measures was 8.8 (min: 3, max: 17). Follow-up rates were 97% (400 cases), 95% (392 cases), 92% (378 cases), 89% (365 cases), and 77% (318 cases) at 12, 24, 36, 48, and 60 months, respectively. Dyslipidemia, hypertension, and sleep apnea prevalence were significantly different between groups (Table 1, P < 0.05).

**Table 1:** The comparison of the patient’s baseline characteristics between T2DM − and T2DM +

	N (%)		P
	T2DM +	T2DM-	
Age < 50 year	55 (89)	274 (81)	
BMI > 45 kg/m^2^	28 (47.5)	156 (47.9)	0.12
Sex			
Male	12 (19.4%)	69 (19.8%)	0.46
Female	50 (80.6%)	279 (80.2%	0.93
Education status			
Illiterate	4(6.4%)	28(8%)	0.28
Elementary	22(35.5%)	112(32.2%)	
Diploma	19(30.6%)	107(30.7%)	
Bachelor’s	12(19.4%)	74(21.3%)	
> Bachelor	5(8.1%)	27(7.8%)	
Married	45 (72.5)	253 (72.5)	0.98
Hypertension	31 (50)	50 (14.5)	0.01
Dyslipidemia	43 (70)	154 (45)	0.001
Hypothyroidism	11 (17.5)	58 (16.5)	0.83
Sleep apnea	17 (27.5)	56 (16)	0.03

The mean BMI of the patients was not significantly different between diabetic (45.09 ± 5.42) and non-diabetic (45.9 ± 5.56) patients (P = 0.28). The mean %TWL was significantly lower in diabetic patients, 12 months postoperatively and after that. The superiority of the mean %TWL curve in non-diabetic patients to the diabetic patients was maintained in 60 months follow-up (Table 2).

**Table 2:** Mean ± SD %TWL of the patients postoperativelyT2DM + and T2DM-

Follow-up	Month	%TWL	%TWL		P value
		overall	T2DM +	T2DM-	
Short term	1	11.21 ± 3.21	11.41 ± 3.63	11.17 ± 3.66	0.62
	3	17.98 ± 4.13	17.86 ± 6.1	18.31 ± 5.11	0.59
	6	25.99 ± 6.03	24.34 ± 5.7	26.29 ± 6.06	0.066
	9	30.23 ± 6.63	27.82 ± 6.8	30.66 ± 6.50	0.002
	12	32.14 ± 7.71	30.68 ± 8.22	33.58 ± 7.54	0.007
	18	34.01 ± 8.45	30.72 ± 8.55	34.6 ± 8.3	0.001
	24	33.69 ± 7.66	29.84 ± 8.94	34.40 ± 7.8	0.001
Midterm	36	32.86 ± 8.29	27.56 ± 9.35	33.09 ± 9.02	< 0.001
	48	30.66 ± 8.65	26.23 ± 9.1	31.48 ± 9.57	< 0.001
	60	28.83 ± 9.01	24.2 ± 8.62	29.73 ± 9.44	< 0.001

The analysis showed that the single-status female patients and those with lower educational levels had a lower mean %TWL; however, none of them was statistically significant (P > 0.05). Patients with lower preoperative BMI (< 50 kg/m^2^) and non-diabetic patients had a significantly higher %TWL over a short and long-term follow-up (P < 0.001). Patients with lower initial BMI (< 50 kg/m^2^) experienced a higher %TWL of %11.68 (%9.36, %13.98) and %12.11 (%7.31, %16.90) over Short-term and long-term follow-up compared with the patients with BMI > 50 adjusted for other factors, Respectively (P < 0.001). Moreover, the mean %TWL was %11.87 (%6.84, %16.9) higher for the non-diabetic patients than the people with diabetes controlling for the other factors (P < 0.001). All the results were adjusted for follow-up time and random effects (Table 3).

**Table 3:** The effect of demographic and clinical factors on TWL% in each weight loss phase

Factor	Short-term (< 36 months)	P	Mid-term (36–60 months)	P
	Effect size* (95% CI)		Effect size* (95% CI)	
Age < 50 year	1.13 (− 2.08, 4.35)	0.52	− 0.62 (− 4.38, 5.11)	0.61
BMI < 50 kg/m^2^	11.68 (9.36, 13.98)	< 0.001	12.11 (7.31, 16.90)	< 0.001
Female	− 1.24 (− 4.35, 2.15)	0.48	− 0.57 (− 6.8, 5.66)	0.73
Lower Undergraduate	− 2.61 (− 5.7, 0.07)	0.078	− 4.81 (− 9.6, 0.16)	0.071
Single	− 1.45 (− 3.18, 0.28)	0.26	− 1.61 (− 7.34, 4.12)	0.47
Non-T2DM	5.11 (1.31, 8.91)	0.001	11.87 (6.84, 16.9)	< 0.001

*Adjusted for time (month), which was highly significant (P < 0.001)

## Discussion

Nowadays, BS has become the best treatment option for morbid obesity. RYGB is an effective and long-lasting treatment for weight loss and comorbidity improvement. Long-term data regarding gastric bypass have been lacking due to the complexity of issues regarding follow-up [21–24]. There is no consensus in the literature indicating which factors can predict success after BS, despite a similarity in the characteristics of the samples in terms of age, sex, preoperative BMI, T2DM, high blood pressure, and dyslipidemia [15, 25]. Therefore, more studies with long-term follow-up should be conducted to investigate these factors' effect on weight loss.

In our study, a 5-years follow-up analysis was performed. Our data demonstrated that the mean age of all patients was 40.1 years with a BMI of 45.78 kg/m^2^, that 80% were female. There was no significant difference among age, BMI and sex between diabetic and non-diabetic patients at the surgery, which shows the homogeneity of these variables. Also, marriage and educational level were not significantly different between diabetic and non-diabetic groups. The present study has strengths and limitations. The relatively high number of patients enrolled in this study is a strength and a prolonged and excellent follow-up rate of 93% for 5 years. Different studies showed the superiority of %TWL as a measurement of choice to explain weight loss because preoperative BMI less influences it [13–15]. The patients’ %TWL was about 26%, 32%, 32% 28% in 6, 12, 32, 60 months follow-up, respectively. Our findings regarding %TWL are similar to those of Pereferrera et al., who reported that most patients could expect to lose 33.6 ± 10 at 36 months after RYGB [26]. Van Rijswijk reported a mean %TWL at 1-year follow-up after LRYGB in a pool data of 8818 patients with a mean of 31.9%TWL [15].

In the study of Junior et al., a progressive loss of excess weight following RYGB was observed along the follow-up periods up to the second year (45%, 64%, 70%, and 73% excess weight loss at 6, 12, 18, and 24 months, respectively) [27]. Our results demonstrated suitable weight loss in short and mid-term follow-up, which has been achieved in many other studies. It can be concluded that RYGB induces excellent weight loss in morbidly obese patients. The %TWL changes showed no significant difference between diabetic and non-diabetic groups until 12 months’ follow-up, but after that, %TWL was significantly higher in the non-diabetic group. The superiority of the mean %TWL curve in non-diabetic patients to the diabetic patients was maintained in 60 months follow-up. In both groups, the weight loss trajectory stopped after 18 months. Sjöström also reported that weight loss with RYGB was maximal at 24 months [4]. In our study, T2DM lead to a lower weight loss compared to non-diabetic patients, which agrees with the literature. In a 4-year follow-up study, Junior et al. found that patients with T2DM had a lower weight loss at 18 months after RYGB versus non-diabetic ones [27]. It may be related to insulin metabolism and patient compliance. Diabetic patients, due to hypoglycemia following drug consumption, eat more sweaty food, which may lead to weight gain. On the other hand, the interactive relation between glucose metabolism, appetite, and basal body metabolism can affect weight changes. The evaluations demonstrated a higher rate of %TWL in male patients in short-term follow-up, which was not different in the long term. It may be related to the psychological and physical characteristics of men. In contrast to our data analysis, Junior et al. In a 4-year follow-up revealed that the male sex was associated with limited success after RYGB .[28] This controversy has been concluded by other reports, as determining the effect of sex is complicated due to the fact that the majority of studies include samples that are made up mostly of women [29–31]. Other reports have concluded this controversy as determining the effect of sex is complicated since most studies include samples that are made up mostly of women [29–31]

The main objectives of BS are to promote a significant and sustainable weight loss to improve or resolve comorbidities and to promote a better quality of life with low rates of preoperative and long-term complications. However, weight loss is not homogeneous in this population, even with technical standardization of the surgery [25, 32]

## Conclusion

RYGB is a standard BS resulting in efficient sixty-month weight loss. However, these effects are dependent on many factors. Based on our results BMI > 50 kg/m^2^ is related to lower TWL in short and mid-term follow-up, but the male sex can induce higher weight loss in the short term. On the other hand, T2DM is associated with poor response in long-term follow-up.

## Data Availability

The data that support the findings of this study are available on request from the corresponding author. The data are not publicly available due to privacy or ethical restrictions.
